# Aids to Physiology

**Published:** 1904-03

**Authors:** 


**Aids to Physiology.** By Peyton T. B. Beale. Pp. 239. London: Baillière, Tindall and Cox. 1903.

This is a revised edition of the old *Aids to Physiology*, by Professor Thompson Lowne, and is one of Messrs. Baillière's well-known series of "Aids to." There is a class of students to whom this book will inevitably appeal—those who strive to "commit to memory" only the absolute minimum required by the examiners. We have great pleasure in informing such that this book does not claim, nor indeed is it, a sufficient guide to any examination that leads to a medical qualification. Many points of difficulty to the student are simply set forth, but we miss many of paramount importance to the medical student, such as Pflüger's law and its explanation. It may be useful to pick up just before an examination as a refresher, but it will be of little use before this anxious period.

